# Antimicrobial Activity of Novel C2-Substituted 1,4-Dihydropyridine Analogues

**DOI:** 10.3797/scipharm.1311-04

**Published:** 2014-01-12

**Authors:** Petra Olejníková, L’ubomír Švorc, Denisa Olšovská, Anna Panáková, Zuzana Vihonská, Katarína Kovaryová, Štefan Marchalín

**Affiliations:** 1 Department of Biochemistry and Microbiology, Faculty of Chemical and Food Technology, Slovak University of Technology, Bratislava, Slovak Republic.; 2 Institute of Analytical Chemistry, Faculty of Chemical and Food Technology, Slovak University of Technology, Bratislava, Slovak Republic.; 3 Department of Organic Chemistry, Faculty of Chemical and Food Technology, Slovak University of Technology, Bratislava, Slovak Republic.

**Keywords:** 1,4-Dihydropyridine derivatives, Antibacterial activity, Antifungal activity, Lipophilicity

## Abstract

The antimicrobial activity of 3-methyl-5-isopropyl (or ethyl) 6-methyl-4-nitrophenyl-1,4-dihydropyridine-3,5-dicarboxylate derivatives was evaluated. Prokaryotes (bacteria) appeared to be more sensitive to their antimicrobial activity than were eukaryotes (filamentous fungi). The best antibacterial activity was shown by derivative **33**, which was able to inhibit the growth of *Mycobacterium smegmatis* (MIC_33_ = 9 μg.ml^−1^), *Staphylococcus aureus* (MIC_33_ = 25 μg.ml^−1^), and *Escherichia coli* (MIC_33_ = 100 μg.ml^−1^). In addition, derivative **4** demonstrated its antibacterial power on the acid-fast bacterial species *M. smegmatis* and on Gram-positive *S. aureus.* Focusing on the structure-activity relationship, it appears that the increase in the substituent bulk at the C2 position improved the antibacterial activity of the set of compounds studied. Derivatives **33** and **4**, carrying 2-cyano-3-oxo-3-phenylprop-1-en-1-yl and allyliminomethyl groups, respectively, showed significantly higher inhibition activities on all tested microorganisms in comparison with the rest of the derivatives. This enhancement was also in good correlation with different log P values (lipophilicity parameter).

## Introduction

The treatment of microbial diseases still remains an important scientific challenge in the field of antimicrobial therapy. Despite a large spectrum of antibiotics and chemotherapeutics available for medical treatment, the increasing resistance of microbial pathogens to these compounds is the reason for the intensive search for novel, antimicrobially active structures. In this context, 1,4-dihydropyridines (1,4-DHP) represent compounds with a wide spectra of biological activity. They belong to a major class of Ca^2+^ channel blockers and some of them are routinely used in pharmacology as drugs for the treatment of cardiovascular diseases, including hypertension [1]. More than 12 commercially available, clinically important drugs such as Amlodipine, Nifedipine, Nimodipine, Felodipine, Isradipine, and Nicardipine containing the 1,4-DHP nucleus have been manufactured and used worldwide [2]. Some studies indicated that not only the L-type of Ca^2+^ channel is blocked by these derivatives and some 6-unsubstituted-1,4-dihydropyridine and 2,6-unsubstituted-1,4-dihydropyridine derivatives were tested as potential N-type calcium channel blockers [3]. Nowadays, numerous papers deal with the various biological properties of 1,4-DHPs including antioxidant [4], vasodilator, bronchodilator, anti-atherosclerotic, anti-aggregation, anti-ischemic, anti-diabetic, and antitumor agents [5, 6]. Concerning the antimicrobial activity of 1,4-DHPs, some derivatives have also been shown to be potent antitubercular agents [7–10] with the ability to inhibit both Gram-positive and Gram-negative bacteria [11, 12]. In this context, the antimicrobial activity of various 1,4-DHP derivatives against *Bacillus subtilis, Staphylococcus aureus, Escherichia coli*, *Proteus vulgaris,* as well as *Mycobacterium tuberculosis* was under investigation [13, 14]. Some studies also have aimed at the antifungal activity of 1,4-DHP derivatives against *Aspergillus fumigates* and *Candida albicans* [15]. Moreover, Lacidipine [16], some 3-chlorophenyl [17], and nitrophenyl 1,4-DHP [18] derivatives are considered to be cytotoxic towards *Trypanosoma cruzi* through respiratory chain inhibition.

Generally, from the point of view of structural chemistry, some necessary conditions for the biological activity of 1,4-DHPs known from the scientific literature are as follows: the presence of an unsaturated 1,4-DHP basic ring with no substitution at the N1 atom, low molecular weight substituents (usually alkyl groups) at the C2 and C6 positions, ester groups at the C3 and C5 positions, and a phenyl ring at the C4 position. Furthermore, the ester groups at C3 and C5 should be non-identical, indicating the fact that the C4 carbon becomes chiral. Ortho and meta substitutions of the phenyl ring at C4 elicit sufficient biological activity by their electronic and steric effects, when compared with unsubstituted or para-substituted derivatives [19,20].

In this work, our effort was focused on the antimicrobial screening of 3-methyl 5-isopropyl (or ethyl) 6-methyl-4-nitrophenyl-1,4-dihydropyridine-3,5-dicarboxylate derivatives and their potential to inhibit the growth of model bacteria and filamentous fungi. The experimental data indicate an increase in the antibacterial activity upon replacement of the C2-positioned substituent with different groups.

## Results and Discussion

### Synthesis of 5-Isopropyl 3-methyl 2-[(allylimino)methyl]-6-methyl-4-(3-nitrophenyl)-1,4-dihydropyridine-3,5-dicarboxylate (4)

**Fig. 1:**
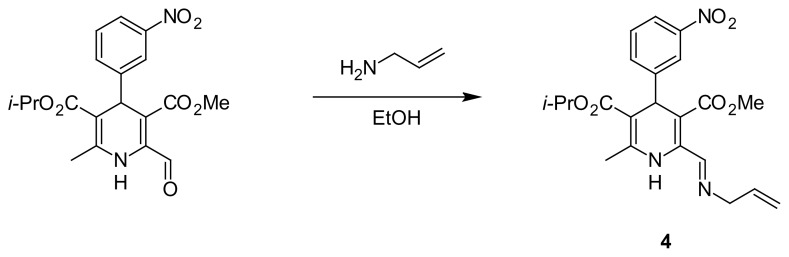
Synthesis of 5-isopropyl 3-methyl 2-[(allylimino)methyl]-6-methyl- 4-(3-nitrophenyl)-1,4-dihydropyridine-3,5-dicarboxylate

To the suspension of 5-isopropyl 3-methyl 2-formyl-6-methyl-4-(3-nitrophenyl)-1,4-dihydropyridine-3,5-dicarboxylate [28] (0.78 g, 2 mmol) in dry ethanol (10 ml), allylamine (0.12 g, 2 mmol) was added and the mixture was refluxed for 3 hours (Fig. 1). After cooling, the solid material was filtered off, washed with ether, dried, and recrystallized from dry ethanol to give **4** in a 81% yield (0.69 g), mp 86–88°C. IR (KBr): ν 3356 (NH), 2978 (CH), 1697 (CO), 1471 (NO_2_) cm^−1; 1^H NMR (CDCl_3_, 300 MHz): δ 1.11 a 1.27 (d, 3H, Me_2_CH; J = 6.3 Hz), 2.43 (s, 3H, Me), 3.71 (s, 3H, MeO), 4.29 (dd, 2H, CH_2_-CH=; J = 1.5 and 6.0 Hz), 4.97 (sept, 1H, OCH; J = 6.0 Hz), 5.18 (s, 1H, H-4), 5.19 (t, 1H, CH=C; J = 1.5 Hz), 5.24 (dq, 1H, CH=C; J = 1.5 and 8.1 Hz), 5.94 – 6.15 (m, 1H, =CH), 7.40 (t, 1H, H-5′; J = 8.1 Hz), 7.64 (d, 1H, H-6′; J = 7.8 Hz), 7.76 (s, 1H, NH), 8.03 (dd, 1H, H-4′; J = 2.4 and 8.1 Hz), 8.14 (t, 1H, H-2′; J = 1.9 Hz), 9.02 (s, 1H, CH=N); ^13^C NMR (75 MHz): δ 19.6 (Me), 21.8 a 22.1 (Me_2_CH), 40.7 (C-4), 51.7 (OMe), 62.3 (N-CH2), 67.4 (Me_2_CH), 102.3 (C-5), 108.7 (C-3), 117.1 (=CH_2_), 121.6 (C-4′), 123.1 (C-2′), 128.9 (C-5′), 134.4 (C-6′), 134.6 (=CH), 139.9 (C-2), 145.1 (C-6), 148.1 (C-3′), 148.9 (C-1′), 154.9 (CH=N), 166.4 (CO_2_), 166.7 (CO_2_); MS, m/z (%): 427(M^+^, 22), 384 (8), 368 (13), 352 (8),340 (16),326 (8),305 (100), 273 (6), 263 (37), 231 (36), 41 (40), 28 (40). Anal. Calcd. for C_22_H_25_N_3_O_6_ (427.5): C, 61.82; H, 5.90; N, 9.83. Found: C, 61.70; H, 5.78; N, 9.11.

### In vitro Antimicrobial Activity and Structure-Antibacterial Activity Evaluation

An *in vitro* antimicrobial assay of the 1,4-DHPs (Tab. 2, Tab. 3) was performed by the dilution method according to the NCLS guidelines on bacteria and filamentous fungi. The results of antibacterial activity are shown in Tab. 2.

**Fig. 2:**
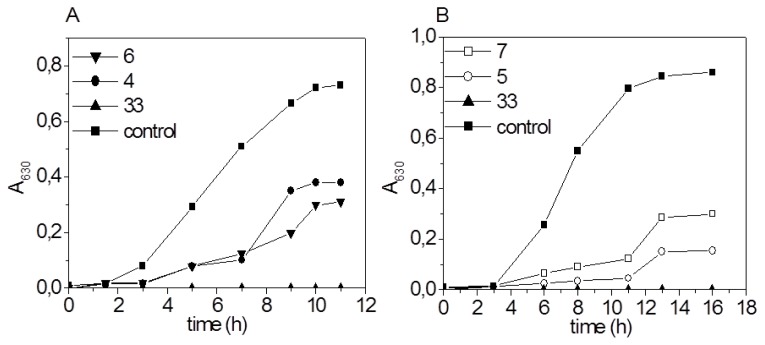
Growth of *M. smegmatis* (A) and *S. aureus* (B) in the presence of 1,4-DHP derivatives in the concentration 25 μg/ml

The best antibacterial activity against *M. smegmatis* was observed in the presence of derivative **33**. In this case, the growth inhibition of *M. smegmatis* was 100% with a bacteriostatic effect on the cells at the concentration of 9 μg/ml. According to our results (Tab. 2), derivative **4** also exhibited good inhibition effects on the growth of *M. smegmatis*. The MIC value of derivative **4** was determined at the concentration value of 50 μg/ml. Fig. 2A shows the growth of *M. smegmatis* in the presence of active derivatives **4**, **6**, and **33** at the concentration of 25 μg/ml. It can be seen from the growth curves that the tested compounds prolong the lag phase of the bacterial growth. In this work, a slight inhibitory effect towards *S. aureus* has also been observed. Derivatives **6** and **U** did not inhibit the growth of the model Gram-positive bacteria (Tab. 2). The illustrative example of the growth inhibition of *S. aureus* in the presence of compounds **33**, **5**, and **7** is depicted in Fig. 2B. A member of the Gama proteobacteria, *E.coli* was inhibited only by derivative **33**. At the concentration value of 100 μg/ml, this derivative displayed growth inhibition of 100% with a bacteriostatic effect on the cells (Tab. 2). According to the results of Albu Mehal [21], 1,4-DHP derivatives showed better activity against Gram-positive than against Gram-negative bacteria, as also confirmed in our work.

**Fig. 3:**
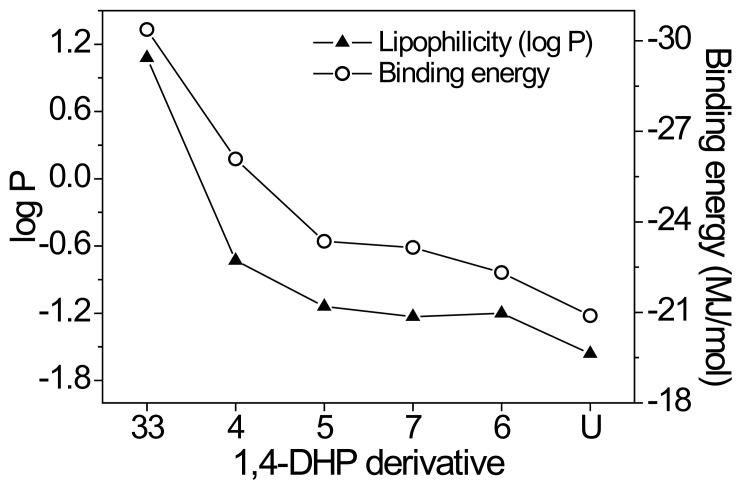
Calculated lipophilicity and binding energy values for 1,4-DHP derivatives studied

It has unambiguously been shown, and also corroborated by the biological activity listed in Tab. 2, that the highest antibacterial activity against all tested bacteria was demonstrated by **33**. Considering its structure, this derivative contains a bulkier substituent (2-cyano-3-oxo-3-phenylprop-1-en-1-yl) at the C2 position of the 1,4-DHP ring. We assume that this substituent may probably enhance the antibacterial activity by increasing the lipophilicity (dominance of hydrophobic interactions). It may also represent an important factor in binding character (binding energy) due to the presence of two electron-withdrawing groups (-CO, -CN). Unlike **33,** derivative **4** provides a lower antibacterial activity, probably due to lower lipophilicity related to the less bulky (allylimino) methyl substituent at the C2 position. In order to verify this assumption, basic quantum-chemical calculations (log P, binding energy) for all 1,4-DHP derivatives studied using AM1 semi-empirical methods were performed using HyperChem version 7.1. The results are summarized in Fig. 3. The calculated Log P value for derivative **33** was 1.08. In comparison with derivative **4** (−0.73), the value for derivative **33** was substantially higher, confirming the abovementioned role of lipophilicity caused by the bulk substituent. This value was also the highest when compared with the other compounds under study containing a less bulkier substituent at the C2 position.

The derivatives **5** and **6** are structurally similar except for the substituent at the C5 position containing an isopropyl and ethyl group, respectively. According to the results listed in Tab. 2, derivative **5** showed higher antibacterial activity than **6**. This fact suggested that the change of the substituent at the C5 position did not have a substantial effect on the lipophilicity. In addition, the derivatives (**33**, **4**, **5,** and **7**) with more significant activity contain isopropyl groups at the C5 position, while lower activity was observed in the case of **6** and **U** with ethyl groups at the C5 position. Based on the presence of similar substituents at other positions (C3, C4, and C6) it can be concluded that the center of lipophilicity may be explicitly associated with the substituent at the C2 position. Therefore, our consideration is based on this concept and is specifically related to the particular set of 1,4-DHP derivatives studied.

For illustrative and comparative purposes, the calculation of binding energy of the molecules of 1,4-DHP derivatives studied has also been performed. It is clear that values corresponded well with those of lipophilicity when e.g. derivative **33** with the highest log P value exhibited the lowest binding energy (−7253 kcal/mol). The similar trend was recorded for all compounds studied as shown in Fig. 3. It is also apparent that lipophilicity values of the compounds studied are in good agreement with binding energies of corresponding molecules. Similarly, it could be said that this kind of energy is related to lipophilicity and antibacterial activity in the particular set of 1,4-DHP derivatives studied.

It has also been observed that the compounds designed as lipophilic precursors were more active than the unmodified polar isosteres, probably due to a better penetration of the compound into the cells. In view of this, it appeared of interest to synthesize some novel 1,4-dihydropyridine-3,5-dicarboxylate derivatives with lipophilic groups. These compounds may act as precursors, and after penetration into the cell wall may lead to the formation of 3,5-dicarboxylate anions by enzymatic hydrolysis [9]. Since the role of Ca^2+^ in bacterial cells is not so well-explained as in eukaryotes, we assume that given the structure of the derivatives, they are potential Ca^2+^ channel blockers. There is a relationship between Ca^2+^ and bacterial gyrase especially in studies with mycobacterial cells. DNA gyrases, topoisomerases in bacterial cells, catalyze DNA supercoiling, relaxation, and decatenation reactions. Karkare et al. have shown evidence for the existence of a Ca^2+^-binding site in the GyrA subunit of *M. tuberculosis* gyrase. Ca^2+^ cannot support topoisomerase reactions in the absence of Mg^2+^, but partial removal of Ca^2+^ from GyrA leads to a modest loss in relaxation activity that can be restored by adding back Ca^2+^. More extensive removal of Ca^2+^ by denaturation of GyrA and dialysis against EGTA results in an enzyme with greatly reduced enzymatic activities and finally, the mutation of the proposed Ca^2+^-binding residues also leads to a loss of activity [22]. These results clearly demonstrated the relationship of Ca^2+^ and gyrase activity and show a possible direction for further studies in the mode of action field of 1,4-DHP as potent gyrase inhibitors. A synergic effect of floroquinolone and amlodipine, a Ca^2+^ channel blocker, on antimicrobial activity has also been shown for *Pseudomonas aeruginosa* [23].

1,4-DHP derivatives exhibited a certain degree of influence on the growth of filamentous fungi. Tab. 2 shows the inhibition effects of the derivatives tested at the concentration of 25 μg/ml and 50 μg/ml, respectively.

1,4-DHP derivatives exhibited only moderate effects on the growth of model fungal strains. Already at the concentration of 25 μg/ml and 50 μg/ml, the derivatives inhibited the growth by 30% and 50%, respectively, but with the increasing concentration of these derivatives, the observed antifungal activity curve remained flat. During the cultivation of *B. cinerea* in the presence of derivative **7**, the morphology of the colony was changed in comparison with the control plate. Subsequently, the morphology of hyphae was studied microscopically. As it is evident from Fig. 4, derivative **7** elicited profound changes in the morphology of hyphal tips accompanied by the release of cytoplasmic content. Similar changes in hyphal tips were described by Hudecová [24] in the presence of nifedipine (basic structure of 1,4-DHPs). Authors have found that the addition of external Ca^2+^ that exceeded the concentration of the blocker partly reversed the morphological changes, so they assumed that the calcium homeostasis perturbation, restriction of the Ca^2+^ influx, is the primary cause of the growth inhibition and the trigger of the morphogenic effect [24].

## Experimental

### Chemicals and Reagents

The tested derivatives were prepared at the Department of Organic Chemistry, Faculty of Chemical and Food Technology, Slovak University of Technology, Bratislava. (Tab. 1). Allylamine for the synthesis was obtained from Sigma–Aldrich Ltd. Steinheim, Germany. For the antimicrobial assay, all solutions were prepared in distilled, sterile water on the day of the experiment, 1,4-DHP was dissolved in DMSO (dimethyl sulphoxide, Sigma–Aldrich Ltd. Steinheim, Germany), growth media Mueller-Hinton broth, Sabouraud-glucose broth, and potato-dextrose agar were obtained from Biolife Srl. (Milano, Italy), Microplates and Petri dishes were purchased from Sarstedt (Germany, Numbrecht, Germany).

### Instrumentation

Melting points were measured on a Boetius micro hot-stage apparatus. The infrared spectra were recorded with a Philips analytical PV 9 800 FT IR spectrometer (KBr). The NMR spectra were recorded on a Varian XR-300 spectrometer in CDCl_3_ using tetramethylsilane as the internal standard. The Correlation Spectroscopy (COSY), Nuclear Overhauser Effect Spectroscopy (NOESY), Nuclear Overhauser Difference, and Nuclear Overhauser Effect Difference (DIFF NOE) techniques were used in the assignment of ^1^H–^1^H relationships and the determination of relative configuration. The Heteronuclear Single-Quantum Correlation Spectroscopy (HSQC) and Heteronuclear Multiple-Bond Correlation Spectroscopy (HMBC) techniques were used throughout for the assignment of the ^1^H–^13^C relationships. Ascending thin-layer chromatography was performed on pre-coated plates of silica gel 60 F 254 (Merck) and the spots were visualized using a UV lamp or iodine vapor. Mass spectral measurements were recorded with the AEI MS 902 S spectrometer (70 eV, electron impact). Condensation of allylamine with the 2-formyl-1,4-dihydropyridine derivative proceeded in anhydrous ethanol and gave the desired imine **4** in 81% yield.

### Procedures

#### Antimicrobial Study in vitro

The antibacterial activity of 1,4-DHP was evaluated by a broth microdilution method in accordance to the NCCLS (National Committee for Clinical Laboratory) guidelines [25] on Gram-positive bacteria *Staphylococcus aureus* CCM 3953, and Gram-negative bacteria *Escherichia coli* CCM 3988 (Czech Collection of Microorganisms, Masaryk University, Brno, Czech Republic) and *Mycobacterium smegmatis* (Collection of Microorganisms, Department of Microbiology and Virology, Faculty of Natural Science, Comenius University, Slovak Republic).

Bacteria were grown in Mueller-Hinton broth overnight at 37°C. For inoculation, the cultures were adjusted to the McFarland No. 0.5 turbidity standard and fresh Mueller Hinton broth was inoculated with the bacterial suspension 1:100. 198 μl of inoculated broth was transferred to each well of the 96-well plate that contained 2 μl of the tested derivatives dissolved in DMSO (final concentrations 100–1 μg/ml). The inoculated microplates were incubated at 37°C until the growth reached the stationary phase. The antimicrobial activity was characterized by the IC_50_ values (concentration of a derivative, which in comparison to the control inhibits the growth of microorganisms to 50%) and MIC values (minimal inhibitory concentration of a derivative which inhibits microbial growth by 100%). These values were read from toxicity curves. The MIC was defined as the lowest concentration of the compound that inhibited the growth.

An antifungal assay was done against filamentous fungi using the agar macrodilution technique [26]. Model filamentous fungi *Aspregillus fumigatus* CCM F-373, *Botrytis cinerea* CCM F-16, and *Alternaria alternata* CCM F-128 were obtained from the Czech Collection of Microorganisms, Masaryk University, Brno, Czech Republic. *Microsporum gypseum* in our experiments was obtained from the collection of microorganisms at the Department of Biochemistry and Microbiology, Faculty of Chemical and Food Technology, Slovak University of Technology, Bratislava, Slovak Republic. Antifungal activity was evaluated on solidified potato-dextrose media during static cultivation. Potato-dextrose agar with the appropriate concentration (100–25 μg/ml) of derivatives tested was inoculated by a fungal conidial suspension (1.10^6^ conidia/ml) in the centre of growth media in Petri dishes. Fungi were cultivated at 25°C for 96 h [26]. The radial growth of the fungal colonies was measured in millimeters. All experiments were performed in three parallels in two independent assays.

#### Computer Modelling

The calculations of log P (lipophilicity parameter, octanol-water partition coefficient) and binding energy (MJ/mol) were carried out using HyperChem version 7.1 (Hypercube, Inc., Gainesville, FL 32608, USA). All of the molecules were drawn into this software and pre-optimized using the MM+ molecular mechanics force field using the Polak–Ribiere algorithm until the root mean square gradient reached 0.01. A more accurate optimization was done with the semi-empirical AM1 method [27].

## Figures and Tables

**Tab. 1:** Set of 1,4-DHP derivatives tested for antimicrobial activity

Cpd. Nr.	1,4-DHP derivative	Structural derivative name [published in]
**4**	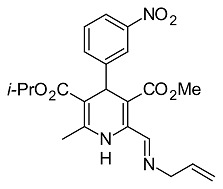	5-isopropyl 3-methyl 2-[(allylimino)methyl]-6-methyl-4-(3-nitrophenyl)-1,4-dihydro-pyridine-3,5-dicarboxylate [This work]
**33**	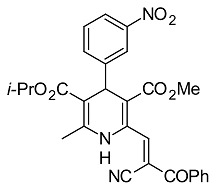	(E)-5-isopropyl 3-methyl 2-(2-cyano-3-oxo-3-phenylprop-1-enyl)-6-methyl-4-(3-nitro-phenyl)-1,4-dihydropyridine-3,5-dicarbo-xylate [28]
**7**	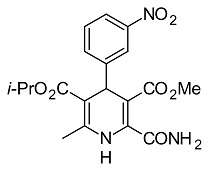	5-isopropyl 3-methyl 2-carbamoyl-6-methyl-4-(3-nitrophenyl)-1,4-dihydropyridine-3,5-dicarboxylate [29]
**5**	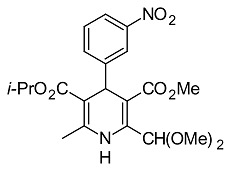	5-isopropyl 3-methyl 2-(dimethoxymethyl)-6-methyl-4-(3-nitrophenyl)-1,4-dihydropyridine-3,5-dicarboxylate [29]
**6**	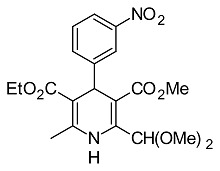	5-ethyl 3-methyl 2-(dimethoxymethyl)-6-methyl-4-(3-nitrophenyl)-1,4-dihydropyridine-3,5-dicarboxylate [29]
**U**	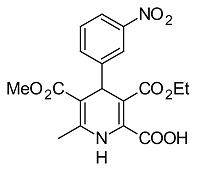	3-(ethoxycarbonyl)-5-(methoxycarbonyl)-6-methyl-4-(3-nitrophenyl)-1,4-dihydropyridine-2-carboxylic acid [30]

**Tab. 2:** Antibacterial activity of 1,4-DHP derivatives expressed by MIC and IC_50_ (μg/ml) evaluated by the broth microdilution method

	model bacteria					
	
	*M. smegmatis*		*S. aureus*		*E. coli*	
	
tested cpd.	MIC	IC_50_	MIC	IC_50_	MIC	IC_50_
**7**	100_s_	75	50_s_	9	> 100	> 100
**33**	9_s_	3	25_s_	9	100s	50
**5**	> 100	56	30_s_	12	> 100	> 100
**6**	100_s_	25	> 100	> 100	> 100	> 100
**U**	> 100	> 100	> 100	> 100	> 100	> 100
**4**	50_s_	31	25_s_	12	> 100	> 100

s…bacteriostatic effect on the bacterial growth.

**Tab. 3:** Growth of model filamentous fungi in the presence of 1,4-DHP derivatives tested at the concentration of 100 μg/ml

	Model fungi				
	
	Growth (%)				
	
Tested cpd.	*A.flavus*	*A.fumigatus*	*A. alternata*	*B.cinerea*	*M.gypseum*
7	80^a^	100	40^b^	70^a^	100
33	70^a^	100	50	100	100
5	100	65	100	80^a^	100
6	100	65	70^a^	100	100
U	100	100	100	100	100
4	70^a^	70^a^	80^a^	100	100

a …Growth inhibition of the fungus was observed at the concentration range of 25–100 μg/ml;

b …Growth inhibition of the fungus was observed at the concentration range of 50–100 μg/ml.
